# SARS-CoV-2-related paediatric inflammatory multisystem syndrome, an epidemiological study, France, 1 March to 17 May 2020

**DOI:** 10.2807/1560-7917.ES.2020.25.22.2001010

**Published:** 2020-06-04

**Authors:** Alexandre Belot, Denise Antona, Sylvain Renolleau, Etienne Javouhey, Véronique Hentgen, François Angoulvant, Christophe Delacourt, Xavier Iriart, Caroline Ovaert, Brigitte Bader-Meunier, Isabelle Kone-Paut, Daniel Levy-Bruhl

**Affiliations:** 1 Filière de santé des maladies auto-immunes et auto-inflammatoires rares (FAI2R), Lyon, France; 2 Santé Publique France, Agence nationale de Santé publique, Saint-Maurice cedex, France; 3 Groupe francophone de réanimation et d’urgences pédiatriques (GFRUP), Paris, France; 4 Groupe de pathologies infectieuses pédiatriques (GPIP), Nice, France; 5 Société Française de Pédiatrie (SFP), Paris, France; 6 Société Française de Cardiologie, filiale de Cardiologie pédiatrique et congénitale (FCPC), Paris, France; 7 Société francophone dédiée à l'étude des maladies inflammatoires pédiatriques (SOFREMIP), Paris, France

**Keywords:** COVID-19, SARS-CoV2, Kawasaki disease, myocarditis, inflammation, children, post-infectious disease

## Abstract

End of April 2020, French clinicians observed an increase in cases presenting with paediatric inflammatory multisystem syndrome (PIMS). Nationwide surveillance was set up and demonstrated temporospatial association with the coronavirus disease (COVID-19) epidemic for 156 reported cases as at 17 May: 108 were classified as confirmed (n = 79), probable (n = 16) or possible (n = 13) post-COVID-19 PIMS cases. A continuum of clinical features from Kawasaki-like disease to myocarditis was observed, requiring intensive care in 67% of cases.

On 28 April 2020, French clinicians alerted the French Public Health Agency about an abnormal increase in cases of Kawasaki-like disease (KLD) and myocarditis in children requiring critical care support that occurred during of the ongoing coronavirus disease (COVID-19) epidemic in France. Concomitantly, Riphagen et al. reported eight children displaying characteristics of hyperinflammatory shock, KLD or toxic shock syndrome [1] and an Italian study reported 10 additional children presenting with a KLD [2].

To investigate this emerging inflammatory disease in children, now named paediatric inflammatory multisystem syndrome (PIMS) or multisystem inflammatory syndrome in children (MIS-C), a nationwide surveillance was launched on 30 April, coordinated by the French Public Health Agency and French paediatric scientific societies. All French paediatric departments were asked to report retrospectively and prospectively all cases of this hyperinflammatory syndrome diagnosed since 1 March to Santé Publique France. 

The objectives of this surveillance were to estimate the burden of PIMS in France, to describe the spatial and temporal dynamics of this emergence in order to investigate its link with the COVID-19 epidemic.

## Description of the surveillance

A reporting form was developed which included age of the patient, results of either RT-PCR or serology for severe acute respiratory syndrome coronavirus 2 (SARS-CoV-2), main clinical features (including seritis, attributes of macrophage activation syndrome (MAS), myocarditis or KLD), type of wards (conventional paediatric unit or intermediate/intensive care unit (ICU)) and, for children admitted to ICU, type of care required (including vasopressor, mechanical ventilation and extracorporeal membrane oxygenation) and if relevant, occurrence of death. No follow-up of the child’s condition was planned in this initial data collection. Whenever either the PCR or the serology was noted as pending, clinicians were subsequently asked by email to update the questionnaire a few days after initial notification.

Based on the main clinical features and on the available information regarding SARS-CoV-2 status, cases were classified into four categories, according to their link with COVID-19:

• Confirmed/proven cases of SARS-CoV-2-related PIMS (CoV-PIMS) were children presenting with one or more of the following symptoms: seritis, characteristics of MAS, myocarditis and/or KLD and a positive SARS-CoV-2 RT-PCR or serology;

• Probable CoV-PIMS cases were children presenting with any of the above clinical features and either a direct epidemiological link with a confirmed COVID-19 case or a chest computed tomography scan favouring the diagnosis of COVID-19;

• Possible CoV-PIMS cases were children presenting with at least two of the above clinical features with pending or not performed PCR and serology;

• Non-CoV PIMS cases were children with both negative PCR and serology or with pending or not performed PCR and serology and presenting with only one of the above clinical features.

We compared the characteristics of the non-CoV PIMS and CoV-PIMS populations using Mann and Whitney test.

The main analysis for CoV-PIMS was performed on possible, probable and confirmed cases only. A comparison of our own case definition with the case definitions from the World Health Organization (WHO), the United States Centers for Disease Control and Prevention (US CDC) and the Royal College of Paediatrics and Child Health (RCPCH) are reported in the Supplementary Table [3-5].

## Findings from the surveillance

**Figure 1:**
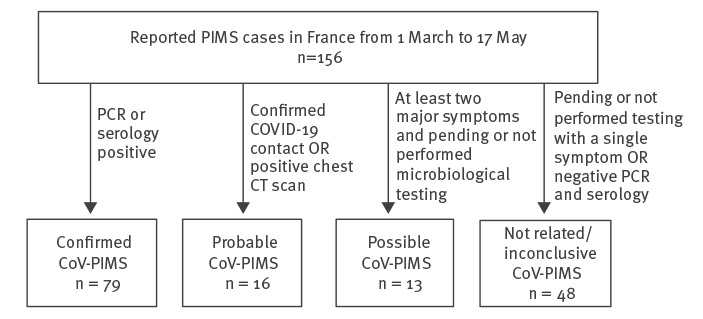
Flowchart of paediatric inflammatory multisystem syndrome cases following classification, France, 1 March–17 May (n = 156)

CoV-PIMS: SARS-CoV-2-related paediatric inflammatory multisystem syndrome; COVID-19: coronavirus disease; CT: computed tomography.

By the end of week 20 (17 May 2020), a total of 156 cases had been notified, 79 classified as confirmed, 16 as probable and 13 as possible CoV-PIMS cases. The 48 remaining cases were ruled out based on our case definition (Figure 1).

**Figure 2:**
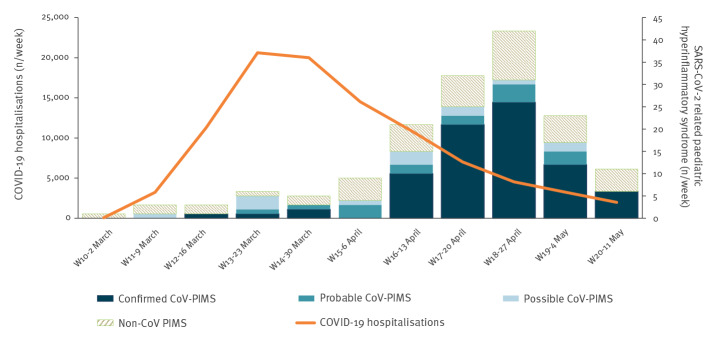
Temporal distribution of COVID-19 hospitalisations and SARS-CoV2 hyperinflammatory paediatric cases, France, 2 March–17 May (n = 108)

CoV-PIMS: SARS-CoV-2-related paediatric inflammatory multisystem syndrome;COVID-19: coronavirus disease; SARS-CoV-2: severe acute respiratory syndrome coronavirus 2.

**Figure 3:**
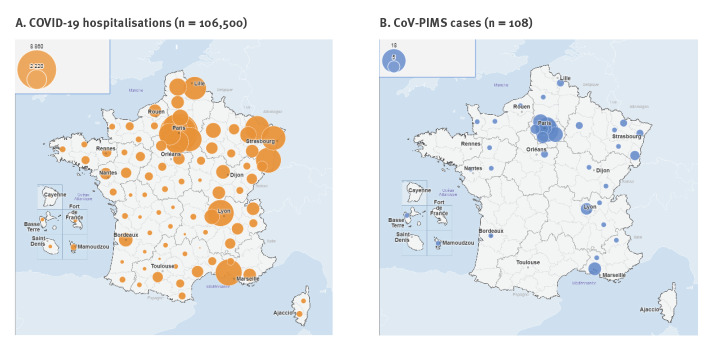
Spatial distribution of COVID-19 hospitalisations and SARS-CoV-2 hyperinflammatory paediatric cases, France 1 March–17 May (n = 108)

CoV-PIMS: SARS-CoV-2-related paediatric inflammatory multisystem syndrome;COVID-19: coronavirus disease; SARS-CoV-2: severe acute respiratory syndrome coronavirus 2.

The epidemic curve of the 108 analysed cases revealed a sharp increase in incidence after 13 April, culminating in week 18, 4–5 weeks after the peak of the COVID-19 epidemic in France and decreasing thereafter (Figure 2). The geographical distribution of cases was comparable to the one of all-ages COVID-19 hospitalisations (Figure 3). Current or past SARS-CoV-2 infection was confirmed by RT-PCR only for 28 cases, by serology only for 42 cases and by both tests for nine cases. 

**Figure 4:**
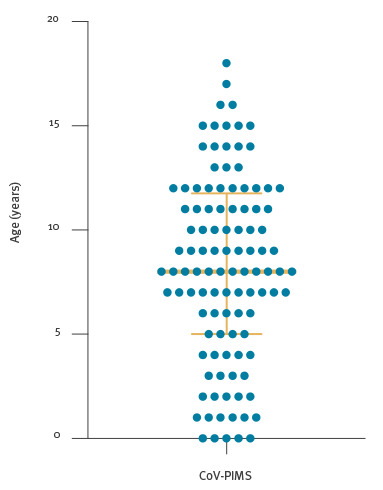
Age distribution of paediatric inflammatory multisystem syndrome patients, France, 1 March–17 May (n = 108)

**Figure 5:**
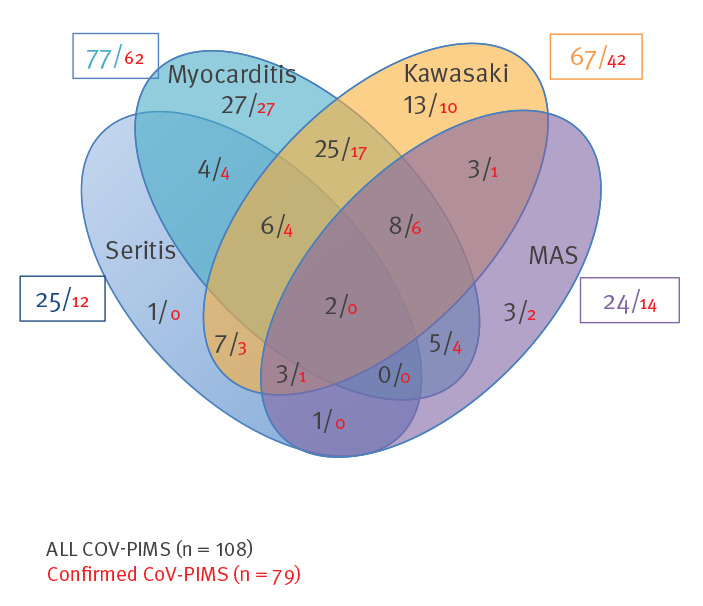
Venn diagram of clinical features of SARS-CoV-2-related paediatric inflammatory multisystem syndrome, France, 1 March–17 May (n = 108)

CoV-PIMS: SARS-CoV-2-related paediatric inflammatory multisystem syndrome;MAS: macrophage activation syndrome.

Age distribution showed a median of 8 years and an interquartile range of 5–11 years (Figure 4). The CoV-PIMS and non-CoV PIMS cases followed a significantly different pattern in the two populations, especially in terms of age distribution, clinical presentation and severity (Table). In CoV-PIMS cases, KLD and myocarditis were the most prevalent clinical features and were associated with 61% and 70% of the cases, respectively. Seritis and features of macrophage activation syndrome (MAS) were also overrepresented with a frequency of 22% and 23% (Figure 5). Critical care support was required in 67% of cases and within this group, 73% required vasopressors and 43% mechanical ventilation. One death was recorded.

CoV-PIMS: SARS-CoV-2-related paediatric inflammatory multisystem syndrome.

**Table:** Comparison of possible, probable and confirmed CoV-PIMS with non-CoV PIMS following our classification criteria, France, 1 March–17 May (n = 156)

	CoV-PIMS (n = 108)		Unrelated CoV-PIMS (n = 48)		p value
Age in years (median; IQR)	8 (5–11)		3 (1–7)		**< 0.0005**
Sex ratio male/female	0.96		1		0.99
Clinical presentation	n	%	n	%	
Kawasaki-like disease	66	61	39	81	**< 0.01**
Myocarditis	76	70	5	10	**< 0.0001**
MAS	25	23	1	2	**< 0.001**
Seritis	24	22	5	10	0.11
Intensive care unit	72	67	4	8	**< 0,0001**

IQR: interquartile range; MAS: macrophage activation syndrome; NA: not applicable.

p values were calculated using the Mann-Whitney test for quantitative values and Fisher’s exact test for qualitative ones.

## Discussion

This study is, to date, the largest series of published PIMS cases, with more than 100 cases. It supports a causal relationship between SARS-CoV-2 infection and PIMS: 95 of the 156 notified cases were confirmed or probable post-COVID cases. Among the 48 excluded cases, 39 presented with KLD symptoms, probably reflecting the classical Kawasaki disease. Our case definition differed slightly from those proposed later on by the WHO, the RCPCH and the US CDC, mainly because we included as a possible case a patient with a pending or not performed diagnosis of SARS-CoV-2 infections. However, we believe that having classified them as possible PIMS cases reflects the actual likelihood of those cases being real PMS cases. Moreover, possible cases only represented 12% of all cases kept in the analysis and their temporal distribution as well as their age distribution and clinical features (data not shown) did not differ from those of probable and confirmed cases. The significant differences between the CoV2-PIMS and non-CoV2 PIMS cases regarding age distribution and main manifestation support a correct classification. We also highlight that further clinical reporting on all manifestations is required to improve the case definition and disease description. 

The epidemic curve of the PIMS cases followed that of COVID-19 with a lag time of 4–5 weeks, supporting the hypothesis of PIMS being a post-infectious manifestation. The geographical distribution of the PIMS cases also correlated with that of the COVID-19 cases. The almost simultaneous detection of PIMS cases in three other places heavily affected by the SARS-CoV-2 epidemic (Italy, the United Kingdom and New York City, US) [6], further reinforces this hypothesis. Conversely, the absence of identified PIMS cases in some countries may reflect (i) a smaller COVID-19 epidemic, (ii) limited awareness of clinicians, (iii) a lack of a specific surveillance system for KLD or other systemic inflammatory symptoms in children, (iv) additional risk factors in our population such as genetic factors or (v) a combination of the above. 

Our study gives some insight into the actual risk of PIMS in children with COVID-19. Indeed, with the help of all concerned learned societies, we were able to set up a specific emergency notification system. We believe that the rarity and severity of the disease with frequent ICU admission, in a context of large media coverage of this new syndrome, has most probably led to a high notification rate. In the absence of specific routine Kawasaki disease surveillance, we were unable to compare the number of notified PIMS cases classified as non-CoV PIMS cases with historical classical Kawasaki disease background rates.

Coronaviruses have previously been reported as a possible trigger of classical Kawasaki disease [7] but represent yearly less than 10% of virus infections associated with classical Kawasaki disease [7]. The older age and the balanced sex ratio in SARS-CoV-2-associated KLD were different from the classical Kawasaki disease which rather occur in the youngest and male children [8,9]. MAS and seritis with systemic inflammation are infrequent in Kawasaki disease and reminiscent of other autoinflammatory diseases [9]. A genetic susceptibility for this post-infectious disease has already been hypothesised [10]. Genetic variation of the virus may be also considered for further exploration. 

In our report, 73% of patients required vasopressor/inotrope support in the ICU and one case was fatal. The European Centre for Disease Prevention and Control (ECDC) Rapid Risk Assessment from 15 May [6] identified six deaths reported globally including the one in France. Early recognition of this syndrome is critical for careful management, especially regarding the occurrence of myocardial dysfunction and shock as highlighted in a first French study [11]. Additional reports also emphasise an increase of other post-infectious diseases such as Guillain–Barré syndrome [12]. Thus, SARS-CoV-2 represents a potent inflammatory trigger in both children and adults. While interferon defect has been reported in critically ill adult patients with severe outcome of the viral infection [13], specific immunological responses in children need further consideration to explore this delayed inflammatory syndrome.

## Conclusion 

French surveillance data confirm the signal of the emergence of an inflammatory multisystem syndrome associated with SARS-CoV-2 infection in children. The actual risk of this disease is difficult to estimate, as reliable data on the incidence of COVID-19 infections in children are not yet available. COVID-19 cases in children younger than 15 years reported to The European Surveillance System (TESSy) represent only 2.1% of all laboratory-confirmed cases. Under the conservative estimate of no more than only 5% of French children under 15 years having been infected with SARS-CoV-2, the risk of PIMS, based on confirmed, probable and possible cases would be fewer than two per 10,000 children. 

In the short term, the risk of new cases of COVID- PIMS is likely to be very low in France, given the low circulation of the virus in France in the past few weeks. More data on this new syndrome will be collected through a research protocol that is currently being implemented. Countries with current high incidence of COVID-19 in the general population should consider this rare but severe delayed syndrome in children.

## Supplementary Data

99000 Supplement
