# Supplementary material for: SARS-CoV-2-related paediatric inflammatory multisystem syndrome, an epidemiological study, France, 1 March to 17 May 2020
Source: Euro Surveill. 2020 Jun 4;25(22):2001010. doi: 10.2807/1560-7917.ES.2020.25.22.2001010 (PMC7336112; doi:10.2807/1560-7917.ES.2020.25.22.2001010)
Supplement: Supplement [file 20-01010_BELOT_Supplement.pdf]

This supplementary material is hosted by Eurosurveillance as supporting information alongside the article [SARS-CoV-2-related paediatric inflammatory multisystem syndrome, an epidemiological study, France, 1 March to 17 May 2020], on behalf of the authors, who remain responsible for the accuracy and appropriateness of the content. The same standards for ethics, copyright, attributions and permissions as for the article apply. Supplements are not edited by Eurosurveillance and the journal is not responsible for the maintenance of any links or email addresses provided therein.

Supplementary Table: Comparison of current case definitions

	CASE DEFINITION			
	French Surveillance<br>(30th April 2020)<br>Pediatric Inflammatory Multisystem Syndrome	WHO<br>(15th May 2020)[1]<br>Multisystem Inflammatory Syndrome in children and adolescents temporally related to COVID-19	US-CDC<br>(14th May 2020)[2]<br>Multisystem Inflammatory Syndrome in Children (MIS-C)	RCPCH<br>(1st May 2020)[3]<br>Paediatric multisystem inflammatory syndrome temporally associated with COVID-19
Inflammation	Fever and systemic inflammation	Fever >3 days AND elevated markers of inflammation (ESR, CRP or procalcitonin)	Fever, laboratory evidence of inflammation	Fever >38.5
Age	0-18 years	0-19 years	<21 years	Child
Main features	At least one of the following MAS, myocarditis, seritis Kawasaki-Like features (if evidence for SARS-CoV2 positive) or at least two items if pending missing virological results	At least Two of the following:<br>-Rash or bilateral non-purulent conjunctivitis or muco-cutaneous inflammation signs (oral, hands or feet).<br>-Hypotension or shock.<br>-Features of myocardial dysfunction, pericarditis, valvulitis, or coronary abnormalities (including ECHO findings or elevated Troponin/NT-proBNP),<br>-Evidence of coagulopathy (by PT, PTT, elevated d-Dimers).<br>-Acute gastrointestinal problems (diarrhea, vomiting, or abdominal pain).	Evidence of clinically severe illness requiring hospitalization, with multisystem (>2) organ involvement (cardiac, renal respiratory, hematologic, gastrointestinal, dermatologic or neurological)	persistent fever, inflammation (neutrophilia, elevated CRP<br>and lymphopaenia) and evidence of single or multi-organ dysfunction (shock, cardiac, respiratory, renal, gastrointestinal or neurological disorder) with additional features. This may include children fulfilling full or partial criteria for Kawasaki disease.
Exclusion criteria	Negative for both SARS-CoV2 related PCR and serology	No other obvious microbial cause of inflammation, including bacterial sepsis, staphylococcal or streptococcal shock syndromes.	No alternative plausible diagnoses	Exclusion of any other microbial cause, including bacterial sepsis, staphylococcal or streptococcal shock syndromes, infections associated with myocarditis such as enterovirus (waiting for results of these investigations should not delay seeking expert advice).
SARS-CoV2 status	Positive for current or recent SARS-CoV2 infection by RT-PCR, serology (confirmed PIMS) or COVID-19 exposure or CT scan evovative (Probable PIMS)<br>Pending or not performed in Possible PIMS (at least two criteria)	Evidence of COVID-19 (RT-PCR, antigen test or serology positive), or likely contact with patients with COVID-19.	Positive for current or recent SARS-CoV2 infection by RT-PCR, serology or antigen test or COVID-19 exposure within the 4 weeks prior to the onset of symptoms	SARS-CoV-2 PCR testing may be positive or negative

ESR: Erythrocyte sedimentation rate, CRP: C reactive protein, US-CDC: United States -Centers for Disease Control and Prevention, MAS: macrophage activation syndrome, RCPCH: Royal College of Paediatrics and Child Health, WHO: World Health Organization,

References:

1. World Health Organization (WHO). Multisystem inflammatory syndrome in children and adolescents temporally related to COVID-19. Geneva: WHO: 15 May 2020. Available from: https://www.who.int/news-room/commentaries/detail/multisystem-inflammatory-syndrome-in-children-and-adolescents-with-covid-19

2. Health Alert Network. Multisystem Inflammatory Syndrome in Children (MIS-C) Associated with Coronavirus Disease 2019 (COVID-19) CDCHAN-00432. Atlanta: Centers for Disease Control and Prevention; 14 May 2020. Available from: https://emergency.cdc.gov/han/2020/han00432.asp

3. Health Policy Team. Guidance - Paediatric multisystem inflammatory syndrome temporally associated with COVID-19. London: Royal College of Paediatrics and Child Health. Available from: https://www.rcpch.ac.uk/resources/guidance-paediatric-multisystem-inflammatory-syndrome-temporally-associated-covid-19 (accessed May 30, 2020).</eref>
